# Design and evaluation of a wedge-shaped adaptive knee orthosis for the human lower limbs 

**DOI:** 10.3389/fbioe.2024.1439616

**Published:** 2024-08-30

**Authors:** Xin Zhou, Xiaoli Liu, Jiaxin Hao, Yu Liu, Yunqi Tang

**Affiliations:** ^1^ School of Art and Design, Xi’an University of Technology, Xi’an, China; ^2^ School of Mechanical and Precision Instrument Engineering, Xi’an University of Technology, Xi’an, China; ^3^ Xi’an Children’s Hospital, The Affiliated Children’s Hospital of Xi’an Jiaotong University, Xi’an, China; ^4^ School of Mechanical Engineering, Xi’an Aeronautical University, Xi’an, China; ^5^ College of Art and Design, Shaanxi University of Science and Technology, Xi’an, China

**Keywords:** KOA, knee joint, mechanical design, knee orthosis, pressure test

## Abstract

**Introduction:**

The incidence of knee osteoarthritis (KOA) is moderately correlated with age and body weight and increases with life span and weight gain, associated with tearing and wearing the knee joints. KOA can adjust the force through the human lower limbs, redistribute the load of the knee joint, reduce the pain, and restore mobility when the arthritis changes are mild. However, most of the existing knee orthosis cannot be adjusted adaptively according to the needs of patients.

**Methodology:**

This study establishes a biomechanical model of the knee joint to analyze the medial and lateral forces acting on the joint. The new adjustable knee orthosis is designed. It applies the principle of four-point bending to apply pressure to both sides of the knee joint, thereby adjusting the varus angle and modifying the medial and lateral forces on the knee joint. Through structural optimization, the prototype of the knee orthosis weighs only 324 g. Utilizing three-dimensional scanning technology, discrete point cloud data of the leg surface is obtained, reconstructed, and processed to create a 3D model of the human leg surface. The design ensures a close fit to the human leg surface, offering comfortable wear. A pressure sensing film system is employed to build a pressure sensing test system, where the knee orthosis is worn on a prosthesis for pressure testing to evaluate its ability to adjust knee joint forces.

**Results:**

The pressure test results demonstrate that the knee orthosis can stably provide an adjustment angle of 0–7° and sustain a maximum force of 10N on both sides of the knee joint over extended periods. A self-developed 8-channel plantar pressure sensing insole is calibrated against commercial plantar pressure sensors. Human wear tests on 15 subjects show that during the operation of the knee orthosis, it significantly adjusts plantar pressures, reducing lateral foot pressures by 22% overall, with more pronounced corrective effects observed in lighter participants.

**Discussion:**

In this study, a wedge-shaped adaptive knee orthosis was provided for KOA patients. The four-point force principle was used to balance the force between femurs and tibia and adjust the meniscus contact gap. The orthotic appliance has the advantages of simple mechanical structure, adjustable correction Angle and good wearing comfort.

## 1 Introduction

Knee osteoarthritis (KOA) is a chronic disease characterized by articular cartilage deformation, abrasion, and bone hyperplasia (Heidari, 2011). Moreover, it is more common in middle-aged and elderly people, and its main physiological manifestations are discomfort symptoms such as knee swelling, pain, and soreness during standing and walking (Louise et al., 2008). In addition to age, lower limb force line deviation, obesity, and trauma were also the main factors affecting the prevalence of KOA (Bliddal and Christensen, 2009). Due to the limitations of medication and surgery, therapists are increasingly recommending the use of accessories, including mechanical insoles and knee orthosis (Xin et al., 2021a).

The orthosis can limit the abnormal movement of the limbs, maintain joint stability, provide certain auxiliary power for the weak parts of muscles, and also restore the movement ability (Folmar et al., 2020). It is an external device for the rehabilitation of motor dysfunction, which can change the functional characteristics or structure of the neuromuscular and skeletal system. It is an important means of orthopedic treatment for bone and joint injury and limb deformity.

For both the KOA patients at an early stage and patients recovering from knee replacement, wearing knee orthosis is an effective way to relieve pain and symptoms. Orthotics are showing increasingly important value in the adjuvant treatment of patients with neuromuscular and skeletal diseases, as well as in the rehabilitation and social reintegration of the disabled. In recent years, the number of disabled people has increased and the demand for assistive devices in the aging population has increased sharply. Many research institutions have carried out a series of studies to form a variety of knee orthoses.

An inflatable three-point force orthosis was designed by Robert D.A. (Gaasbeek et al., 2007). The adjustment of the orthopedic angle was achieved by controlling the degree of inflation of the flexible air bladder. Meanwhile, Mokhtar A designed an inflatable four-point force orthosis (Arazpour et al., 2013). The surface of the human body frequently rubs against the airbag, and the air bladder is easily worn and failed. Cynthia H used the knee joint ectropion orthosis produced by Otto Bock to change the orthopedic angle by adjusting the adjustment screw of the hinge position. However, the adjustment of knee orthosis auxiliary angle needs to rely on specific instruments, and it is inconvenient to adjust the specific auxiliary angle (Cynthia et al., 2009). According to this principle, a new modular self-centering four-point force knee orthosis was designed by Karimi MT (Esrafilian et al., 2012). A new four-point bending partial unloading orthosis was designed by Hangalur et al. (2017), which increases the medial compartment clearance and reduces pain. It is very comfortable to wear but increases cartilage and meniscus wear in the opposite knee joint. Razaei developed a dynamically adjusted orthotic device consisting of inflatable knee pads and insole (Rezaei et al., 2019). The valgus torque in the orthosis was adjusted by using the change of the plantar pressure, but the system was complicated.

The objective of the present study was to achieve the following research goals: 1) enhancing the overall wearing experience for patients while simultaneously reducing the weight of the orthosis, 2) simplifying the mechanical structure to improve practicality, 3) incorporating a fast multi-level adjustment capability to address individual patient requirements. This study introduces a knee orthosis that facilitates adjustments of the force line with the lower limbs and the knee varus angle through fast multi-level adjustments in the coronal plane of the knee joint. By modifying the load distribution within the internal and external compartments of the knee joint, this orthosis aims to alleviate patient pain and restore mobility.

Structural optimization design is a critical research area in modern structural design, providing tools for achieving optimal new structural designs and becoming a significant component of contemporary design methodologies. It involves parameterizing relevant variables according to design requirements to generate a domain of all potential structural design solutions. Mathematical techniques are then used within this domain to search for designs that not only meet predefined criteria but are also feasible and optimal. With the rapid advancement of computer technology and the widespread application of numerical computation methods, optimization capabilities, and computational speeds have correspondingly improved.

For novel knee orthotic structures, which often involve complex and numerous continuum structures with significant shape-dependent performance implications, structural analysis poses certain challenges. Structural optimization design benefits from strong convergence, high reliability, and stability in optimization algorithms, which are continually undergoing development and refinement by researchers. Introducing theories and methods from various modern disciplines, particularly artificial intelligence, neural networks, fuzzy mathematics, uncertainty mathematics, genetic algorithms, and others, has opened new avenues for advancing optimization algorithms. Examples include genetic algorithms, neural network-based algorithms, ant colony algorithms, simulated annealing, and others. In recent years, a plethora of new and excellent algorithms have emerged, propelling the development of design disciplines. These include global optimization algorithms (Younis and Dong, 2010a), novel sampling search methods (Safari et al., 2015), adaptive multi-objective optimization methods based on surrogate models (AMOS) (Younis and Dong, 2024), computational methods using weighted ensemble of surrogates (WESO) (Younis and ElBadawy, 2022), Pareto finder MOO algorithm for automatic multi-objective surrogate (AMSP) (Younis and Dong, 2022), new global optimization algorithms (MSMDSES) (Younis and Dong, 2012), spatial exploration and single-peak region elimination (SEUMRE) algorithm (Younis and Dong, 2010b), approximate single-peak region elimination method (AUREM) (Younis et al., 2009), pursuit sampling optimization algorithm (TR-MPS) (Cheng et al., 2012), and the Grey Wolf Optimizer (GWO) algorithm (Younis et al., 2022). The theoretical framework of structural optimization design has also rapidly evolved, progressing from size optimization to shape, topology, layout, and type optimization; from single-objective to multi-objective optimization; from deterministic to uncertainty-based optimization; and from static structural optimization to dynamic structural optimization, continually elevating the hierarchy of optimization design.

The rest of the paper was organized as follows: Section 2 presents biomechanical properties of the knee joint. Including physiological structure of knee joint, KOA pathological characteristics, prevention, and treatment. And also the mechanical model of the knee joint. Section 3 presents the mechanical structure design and working principle. Section 4 describes the mechanical and functional experiment. Section 5 summarizes the main research results of this paper.

## 2 Biomechanical properties of knee joint

The knee joint belongs to a synovial joint, which is composed of the distal femur, proximal tibia, and patella. It can also be divided into the patella joint and femoral tibia joint. The femoral patella joint contains the femur and patella (Özkaya et al., 2012). Through the joint surface matching and soft tissue constraint balance, the knee extensor muscle strength plays a role in transmission and constraint (Masouros et al., 2010). Femoral-tibial joint, including femur and tibia, is the main joint that determines the kinetics of the knee joint. It mainly relies on the meniscus and related ligament tissue to impose additional constraints on the joint to maintain stability (Reeves Neil and Bowling, 2011). Therefore, it is necessary to analyze the biomechanics of the knee joint before any mechanical structure design aiming to obtain the expected ideal effect.

### 2.1 Physiological structure of knee joint

The essence of medial compartment knee osteoarthritis lies in the alteration of lower limb alignment, commonly manifested as tibial deviation towards the medial side relative to the femur. This alteration results in excessive pressure on the medial compartment, leading to pain and joint deformities. Therefore, in response to this cause, orthotic devices are frequently employed in clinical practice to correct the alignment of the joint, alleviate symptoms, and prevent joint deformities. There are three commonly utilized approaches: lateral wedge insoles, genu varus orthosis, and mechanical corrective footwear.

**FIGURE 1:**
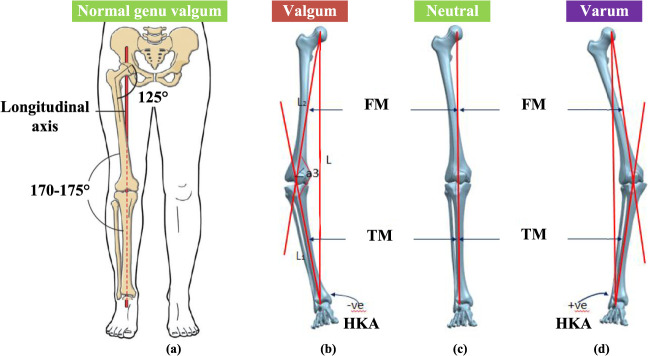
Human lower limb force line. **(A)** Straight line connecting center of femoral head, knee and ankle joint. **(B)** The genu varum of knee joint. **(C)** The neutral knee joint. **(D)** The genu valgus of knee joint. LBA is the lower limb mechanical load line, FM is the femoral axis, TM is the tibial axis.

The knee joint is the largest and most complex joint in the human body in daily activities, the contact force between the tibia and femur is several times the body weight (Taylor et al., 2004). Due to such a large load, the probability of knee injury is higher than other joints. The hip-knee-ankle angle (HKAA) is a common parameter measured using full-length lower limb radiographs, often used to indicate the degree of deformity in knee osteoarthritis (KOA). It is defined as the angle formed at the intersection of lines drawn from the center of the femoral head to the center of the tibial plateau and from the center of the talus to the center of the tibial plateau. As depicted in Figures 1A, in individuals without deformity, HKAA is approximately 180° as shown in Figure 1C. An HKAA angle greater than 180° indicates a varus deformity, while an HKAA angle less than 180° indicates a valgus deformity.

KOA patients are accompanied by a certain degree of genu varus, also known as bow-leg or knock-knee. In patients with knee varus as shown in Figures 1B, degenerative changes can occurr in the knee cartilage, and the mechanical axis of the lower limb shifts to the medial side of the joint. The medial pressure of the joint increase, which aggravates the degree of varus (Cooke et al., 2007). The lateral compartment space of the knee joint is larger than that of the medial compartment, which leads to the shift of the force line of the lower limb to the medial. The medial articular surface is more prone to stress concentration and accelerates the wear of the medial cartilage, resulting in the medial compartment KOA. The mechanical axis of knee valgus patients shifted to the lateral joint as shown in Figure 1D, and the lateral pressure of the joint increases, which aggravates the degree of valgus and the wear of lateral cartilage. Therefore, studying the biomechanical changes of knee cartilage and meniscus is of great significance to the pathogenesis, prevention and orthosis design of early KOA. The incidence rate is higher than that of the lateral compartment.

### 2.2 Knee osteoarthritis (KOA)

Knee osteoarthritis is a common clinical disease. Its pathological characteristics are primary or secondary lesions of articular cartilage. It is a chronic knee joint disease characterized by chronic degeneration, wear and bone hyperplasia of articular cartilage. It is often affected by middle-aged and elderly people. With the increase of age and the decrease of exercise activities, muscles, tendons, ligaments and other soft tissues in human joints are prone to degenerative diseases, resulting in poor coordination of movements, resulting in a high incidence of knee osteoarthritis in the elderly.

#### 2.2.1 KOA pathological characteristics

Primary cartilage lesion of knee joint refers to naturally occurring cartilage lesion without external stimulation; Secondary cartilage lesions of knee joint refer to cartilage lesions caused by joint injuries, such as ligament tear, meniscus tear, synovitis, and other diseases. It is caused by a variety of factors, including age, obesity, trauma, genetics, and so on (Losina et al., 2012). The result is an asymmetric load between the medial and lateral compartments of the knee, causing joint pain (Jackson et al., 2004).

In a normal human gait cycle, knee joint pressure is closely related to KOA pathological process (Hunt et al., 2006). The lower limb load will show periodic changes with the movement of the lower limb. In the moment of foot contact, the load borne by the knee joint can reach 2–3 times the weight force, including the muscle force generated by muscle contraction, self-weight, and the sum force formed by dynamic load (Pandy and Andriacchi, 2010). When the foot strikes the ground during the whole standing period, due to the ground reaction force, the knee joint in the coronal plane will produce a small knee valgus moment. As the gait proceeds, the knee adduction moment (KAM) of greater amplitude and longer duration endures, causing the tibia to rotate inwards to the femur on the coronal plane (Hunt et al., 2008).

When the knee joint is varus or valgus, the angle between the femur and the tibia will change accordingly, and the lower limb force line will deviate to the other side of the knee joint center. As the space between the lateral ventricles of the knee is larger than that of the medial side, the force line of the lower limbs is shifted to the medial side, and the medial articular surface is more prone to stress concentration, which accelerates the wear of the medial cartilage, leading to the majority of the medial ventricles in KOA, with a higher incidence than the lateral (Thorp et al., 2006). The degenerative changes of the cartilage of the knee joint caused by the patient led to the deviation of the mechanical axis of the lower limb to the inside, increasing of adduction moment (Shull et al., 2011).

#### 2.2.2 KOA prevention and treatment

The main treatment methods for KOA include surgical treatment and non-surgical treatment. Currently, surgical treatment is generally used for patients with severe KOA, while conservative treatment is generally used for patients with mild KOA (Hartofilakidis et al., 2014). With the development of medical rehabilitation equipment, lower limb medical rehabilitation exoskeleton technology is becoming more mature. For patients with early and middle-stage KOA, patients who prefer conservative treatment can use knee orthosis for conservative treatment. The basic purpose of conservative treatment of KOA is to relieve pain, maintain joint stability, improve joint function, inhibit cartilage wear and delay joint degeneration.

The use of orthotic insole or knee orthosis can adjust the line of force and increase the space of the joint compartment. By adjusting the angle of the knee joint, it can adjust and balance the force of the articular cartilage and the damaged part of the meniscus, thereby relieving pain, assisting patients to exercise normally, and preventing the deterioration of KOA. As a result, there is a great demand for knee orthosis.

### 2.3 Mechanical model of knee joint

**FIGURE 2:**
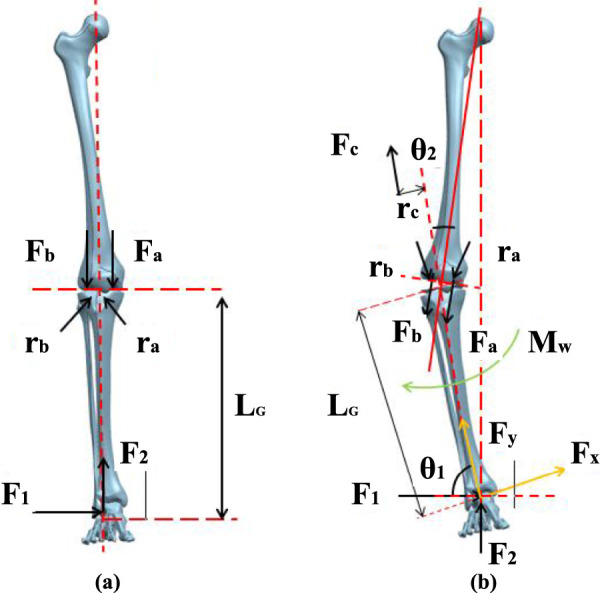
Force line model of human lower limb. **(A)** Normal force line of human lower limb. **(B)** Human lower limb force line in patients with varus.

A simplified force model of the human lower limb is employed to conduct an analysis of the forces acting upon a normal knee joint, as depicted in Figures 2A. In the context of flexion/extension motion in the human knee joint, the axis of motion can be regarded as the bilateral posterior superior condyles located at the distal femur, with the rotational center designated at the intersection of the knee joint axis (Shelburne et al., 2008). The line of force within the lower limb of an average individual passes through the center of the femoral head, the center of the knee joint, and the center of the ankle joint.

During a state of rest, the foot encounters a forward reaction force $F_{2}$ exerted by the ground, as well as a horizontal force $F_{1}$ . The distance from the knee joint center to the ground is denoted as $L_{G}$ . The medial compartment of the knee joint undergoes a positive pressure $F_{a}$ , situated at a horizontal distance ra from the knee joint center, whereas the lateral compartment experiences a positive pressure $F_{b}$ at a horizontal distance $r_{b}$ from the knee joint center. By taking moments at the knee joint center, the equilibrium equation can be formulated as shown in Equation 1:

$$\left\{\begin{array}{l} F_{2}=F_{a}+F_{b}\\ F_{b}\cdot r_{b}-F_{a}\cdot r_{a}+F_{1}\cdot L_{G}=0 \end{array}\right.$$ (1)

The distance between the center of the knee joint and the center of action points in the medial and lateral compartments is approximately equal and is estimated to be one-fourth of the knee width W . The force in the medial and lateral compartments of the knee joint can be achieved as Equation 2:

$$\left\{\begin{array}{l} F_{a}=\frac{F_{2}}{2}+\frac{2F_{1}\cdot L_{G}}{W}\\ F_{b}=\frac{F_{2}}{2}-\frac{2F_{1}\cdot L_{G}}{W} \end{array}\right.$$ (2)

Through the above equation, it can be observed that in order to maintain an upright posture, the medial compartment of the knee joint experiences greater force compared to the lateral compartment, making the medial meniscus more prone to wear and tear. For patients with knee genu varus (inward rotation), the abnormal alignment of the lower limb causes a change in the angle between the tibia and femur centerlines, resulting in a shift in the direction of plantar pressure. Taking the case of a patient with knee genu varus as an example, a lower limb biomechanical model needs to be reconstructed, considering the offset angle generated by internal and external rotation of the knee joint. In order to achieve an overall balance of forces on the knee joint, the knee joint will experience tension from the lateral collateral ligament, as shown in Figure 2B.

The tibial inclination Angle is defined as $\theta_{1}$ . In the coronal plane, the ground reaction force $F_{1}$ and $F_{2}$ decomposed into the tibia are $F_{x}$ and $F_{y}$ , respectively as shown in Equation 3:

$$\left\{\begin{array}{l} F_{x}=F_{1}\sin\theta_{1}+F_{2}\cos\theta_{1}\\ F_{y}=F_{2}\sin\theta_{1}-F_{1}\cos\theta_{1} \end{array}\right.$$ (3)

At this point, the distance from the distal center of the tibia to the point of force application on the sole is $L_{G}$ . The direction of the force on the lateral and medial sides of the knee joint will vary with internal or external rotation of the knee. It points towards the center of the hip joint along the distal center of the femur. Once the knee joint undergoes internal rotation, the lateral compartment will experience tension from the lateral collateral ligament $F_{c}$ . Taking the moment about the center point attached to the lateral collateral ligament as shown in Equation 4:

$$F_{y}r_{c}+F_{x}L_{G}-F_{b}r_{b}\cos\theta_{2}-F_{a}\left(r_{a}+r_{c}\right)\cos\theta_{2}=0$$ (4)

The distance from the lateral collateral ligament to the center of the knee joint $r_{c}$ is approximately equal to half of the knee width W . The force in the medial and lateral compartments of the knee joint can be achieved as Equation 5:

$$\left\{\begin{array}{l} F_{a}=\frac{F_{y}}{\cos\theta_{2}}+\frac{4F_{x}L_{G}}{\text{Wcos}\theta_{2}}-\frac{F_{x}}{2\sin\theta_{2}}\\ F_{b}=\frac{3F_{x}}{2\sin\theta_{2}}-\frac{F_{y}}{\cos\theta_{2}}-\frac{4F_{x}L_{G}}{\text{Wcos}\theta_{2}} \end{array}\right.$$ (5)

When a patient uses an orthosis to correct knee genu varus, it is equivalent to applying an external moment $M_{w}$ of abduction at the knee joint. Taking the moment about the center point attached to the lateral collateral ligament as shown in Equation 6.

$$F_{y}r_{c}+F_{x}L_{G}-F_{b}r_{b}\cos\theta_{2}-F_{a}\left(r_{a}+r_{c}\right)\cos\theta_{2}-M_{w}=0$$ (6)

So the force in the medial and lateral compartments of the knee joint can be achieved as Equation 7:

$$\left\{\begin{array}{l} F_{a}=\frac{F_{y}}{\cos\theta_{2}}+\frac{4F_{x}L_{G}}{\text{Wcos}\theta_{2}}-\frac{F_{x}}{2\sin\theta_{2}}-\frac{M_{w}}{\text{Wcos}\theta_{2}}\\ F_{b}=\frac{3F_{x}}{2\sin\theta_{2}}-\frac{F_{y}}{\cos\theta_{2}}-\frac{4F_{x}L_{G}}{\text{Wcos}\theta_{2}}+\frac{M_{w}}{\text{Wcos}\theta_{2}} \end{array}\right.$$ (7)

By comparing Equations 5, 7, it can be observed that when using a knee orthosis, the presence of the moment $M_{w}$ can reduce the force on the medial compartment of the knee joint $F_{a}$ and increase the force on the lateral compartment to some extent $F_{b}$ . However, considering factors such as the body’s tolerance and the comfort of wearing the knee orthosis, the adjustment of the degree of knee genu varus correction through the application of an external moment $M_{w}$ is limited. It can only alleviate further joint wear and extend the lifespan of the meniscus while correcting the individual’s walking gait.

## 3 Design and working principle

The existing knee orthosis on the market generally fixes the angle of the knee joint in the coronal plane, and cannot adaptively follow the normal motion of the knee joint in the sagittal plane. It can reduce the wear of the joint and relieve the pain of the patient’s knee after the knee joint is fixed with knee orthosis. However, long-term wear can lead to atrophy of the flexion and extension muscles in the knee joint, resulting in the imbalance of muscle strength in the later stage, which affects normal walking.

In order to enhance patients’ experience of wearing knee orthosis, designers will customize the adjustment mechanism of orthoses independently. However, in the process of patients’ lower limb function recovery and knee rehabilitation, the adjustment effect cannot be adjusted adaptively. For example, at the initial stage of wearing, patients refuse to wear knee orthosis due to the high intensity of correction and strong pressure on limbs, which reduce the effect of rehabilitation treatment. In the later stage of rehabilitation, the body’s adaptive adjustment function will adapt to the effect of auxiliary adjustment structure on the human body, forming physiological dependence on the knee orthosis, resulting in long-term wearing and affecting the normal life of patients. In view of the current situation of most knee orthosis, it is particularly important to design a knee orthosis that can adjust the effect of knee varus according to the patient’s own condition.

### 3.1 Mechanical design

**FIGURE 3:**
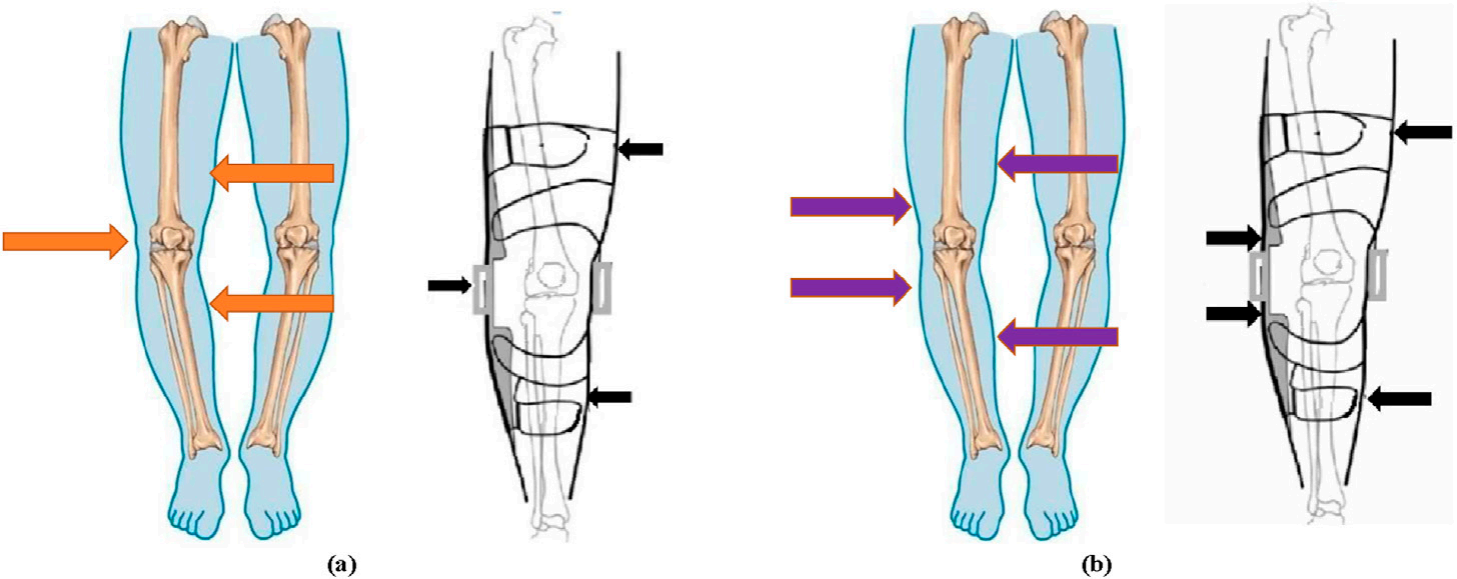
The working principle of knee orthosis. **(A)** The principle of the three-point force formula. **(B)** The principle of the four-point force formula.

The knee joint is in the reciprocating cycle of load-bearing and non-load-bearing in the human gait cycle, and the traditional three-point force and four-point force orthoses usually have good adjustment effects. The principle of three-point force is that the body generates a counterforce against the force exerted by the orthosis, and the two maintain a relative balance to give the wearer continuous pressure (Aleksandra et al., 2020; Khosravi et al., 2019). A force is applied to the knee joint on the thigh and calf of the affected side, and a reverse force is applied to the contralateral condyle as shown in Figure 3A. By applying the overturning moment on the coronal plane of the knee joint, the partial inward torque is offset, to correct the force line of the lower limb.

The principle of the four-point force formula is the same as that of the three-point force formula, which corrects the lower limb force line through torques with opposite directions and different action points. The difference is that the force point is applied to the upper and lower two points of the opposite condyle as shown in Figure 3B. Force was applied on both sides of the knee joint to form a rollover torque to adjust the force line of the human lower limb.

**FIGURE 4:**
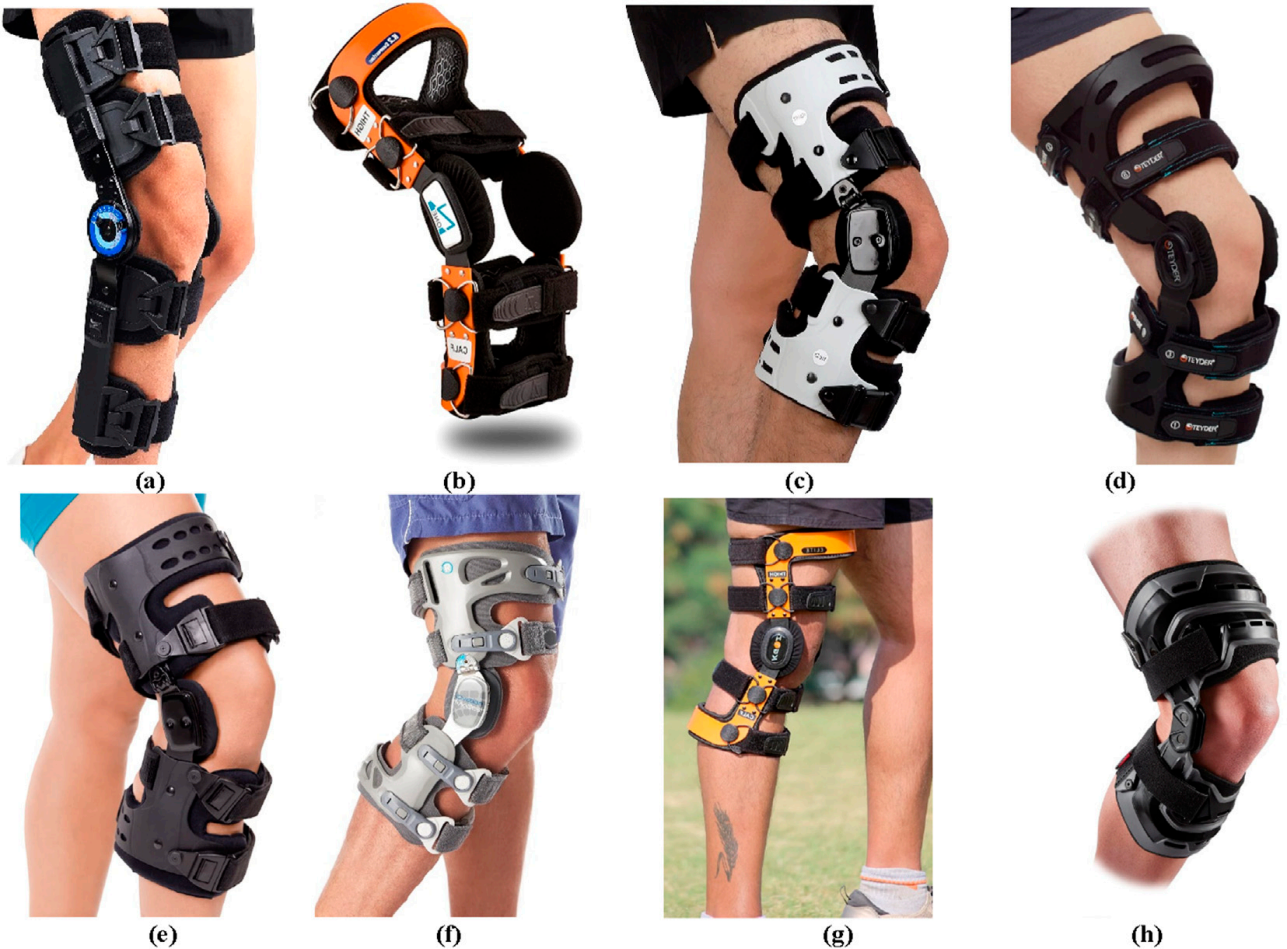
The commercial knee orthotics. **(A)** Orthomen Hinged ROM Knee Brace. **(B)** Z1 K2 Comfortline Hinged Knee Brace. **(C)** Orthomen OA Unloader Knee Brace. **(D)** Teyder functional Knee Brace (476RDR). **(E)** BraceAbility Osteoarthritis Unloader Knee Brace. **(F)** Game Changer Unloader Knee Brace. **(G)** ELITE Knee Support Brace. **(H)** McDavid Heavy Duty Knee Brace.

Through searching and researching the currently available knee orthosis products in the market, it was found that this type of product is diverse and varied in styles. Currently, most commercial knee orthoses employ passive designs, with an average retail price of around USD 120. According to sales volume data, knee orthosis products demonstrate broad market demand. Several representative products were selected based on the principles of three-point and four-point force application, as depicted in Figure 4. Specific product parameters were collected for comparative analysis, as shown in Table 1.

**TABLE 1:** The empirical formula of the human body parameter.

Name	Weight/kg	Main structural material	Function	Adjustability
Orthomen Hinged ROM Knee Brace	1.50	Aluminum	Improve the postoperative knee stability/Control motion amplitude	Telescopic adjustable ROM hinge
Z1 K2 Comfortline Hinged Knee Brace	0.59	Silicone neoprene/Stainless steel	Prevent sports injury/Prevent joint degeneration in mild KOA patients	Condoyles liner adjusts for tightness and comfort
Orthomen OA Unloader Knee Brace	0.84	Polymer plastic	Delay the operating time/Reduce the knee osteoarthritis pain	Adjustable hinge/Adjustable straps
Teyder functional Knee Brace	NA	Aluminum	Improve knee stability/Reduce movement impact	Adjust and fix the knee flexion Angle
BraceAbility Osteoarthritis Unloader Knee Brace	0.66	Polymer	Delay the operating time/Reduce the knee osteoarthritis pain	Adjustable range of motion extension plug-in
Game Changer Unloader Knee Brace	0.54	Neoprene/plastic	Improve knee stability/Reduce knee osteoarthritis pain	Knee flexion and extension Angle adjustment
ELITE Knee Support Brace	1.67	plastic	Reduce knee osteoarthritis pain/Reduce knee load	Customized knee pads without adjustment
McDavid Heavy Duty Knee Brace	0.52	Aluminum	Improve knee support and stability	The strap provides adjustable range and structural support

**FIGURE 5:**
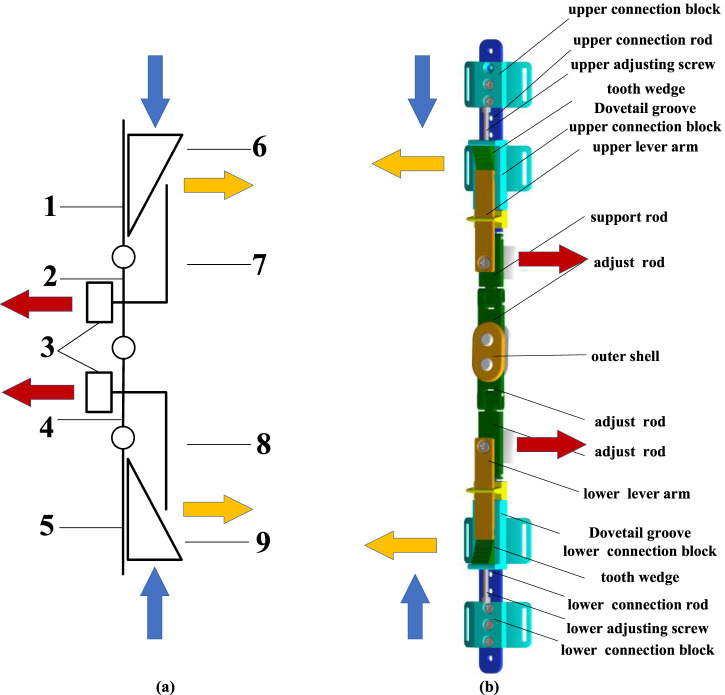
The structure and working principle. **(A)** Diagram of mechanism. 1-upper connecting rod, 2-upper lever support rod, 3-knee joint stress adjustment support brackets, 4-lower lever support rod, 5-lower connecting rod, 6-upper toothed wedge block, 7- upper lever arm, 8- lower lever arm, 9-lower toothed wedge block. **(B)** Assembly view of the knee orthosis.

The new adjustable orthosis for reducing the medial compartment load was designed based on the principle of the four-point force formula (Xin et al., 2021b), as shown in Figure 5A. In order to transfer the load of the affected knee to the contralateral knee to reduce the pressure of the affected knee. The toothed wedge blocks (6, 9) are moved to the center of the knee joint by the screw thrust, and the lever arms (7, 8) are raised to the outside of the leg during the movement. At the same time, the other end of the lever arm (2, 4) support rod squeezes inside the leg, so that the knee joint adjusts the stress brackets (3) to exert sustained pressure on the knee joint.

The main structure of the orthosis, as shown in Figure 5B is composed of connecting rod, upper and lower tooth adjustment toothed wedge blocks, joint flexion connecting rod, and thigh and calf fixed braces. The two ends of the upper and lower knee joint flexion link are incomplete gears, which ensure the flexion motion of the knee joint through tooth meshing. A series of threaded holes are reserved along the vertical direction of the upper and lower connecting rods of the orthosis, which are matched with the upper and lower universal connecting blocks, and the patients can adjust to the appropriate position according to their own conditions. With the cooperation of ratchet and ratchet bar, it has the characteristics of one-way transmission, which leads to the locking of toothed wedge blocks and can’t reverse, so as to ensure the stable correction effect of knee orthosis.

**FIGURE 6:**
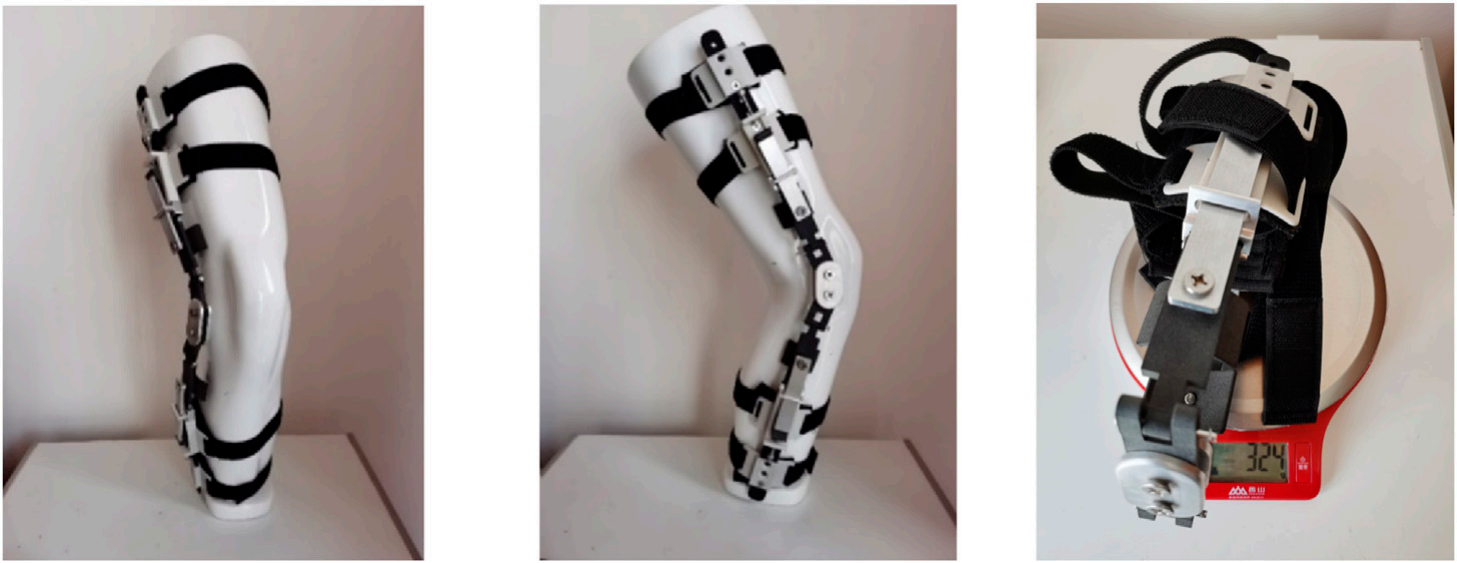
The knee orthosis was worn on the human prosthetic leg model.

The knee orthosis, designed based on the biomechanics of the human lower limb, exhibits the following characteristics. Firstly, employing the principles of mechanical leverage, enables adjustment of the pressure on the knee joint in the coronal plane, thereby modifying the load distribution between the internal and external compartments. This adjustment relieves patients’ pain and restores their mobility. Secondly, the inclusion of a specifically designed hinge allows for optimal alignment with the knee joint’s range of motion, minimizing interference during natural knee movements. Thirdly, the orthosis can be customized to meet the corrective needs of patients at different stages of rehabilitation. Upon assembly, the knee orthosis was fitted onto a human prosthetic leg model, as depicted in Figure 6. Overall, the orthosis demonstrates a straightforward mechanical structure and provides an excellent fit with the human leg. Furthermore, it can be concealed beneath clothing, resembling the appearance of a normal leg, alleviating psychological burdens for the patient. Simultaneously, the special hinge design ensures the patient’s knee joint swings naturally, without impeding regular bodily movements.

### 3.2 Wearable structure design

From the perspective of product design, it is important to prioritize functionality while aiming to achieve an optimal user experience and a comfortable wearing experience. When designing wearable mechanisms for the human body, two key factors should be considered based on ergonomics: materials and structure. On one hand, for wearable products on the human body, there will be interactive forces between the human body and the device during walking or movement. If rigid materials are used exclusively, it may cause secondary harm to the muscles and bones. On the other hand, scientific and rational design is crucial because exoskeletons exert significant pressure and have a large contact area with the human body surface. If the pressure distribution is uneven, it can hinder blood circulation, leading to ischemia and necrosis of soft tissues. If the contact area is not properly designed, it can impede skin respiration and prevent sweat from evaporating, eventually resulting in skin ulcers.

Drawing upon the structure and characteristics of the human lower limb, a compatible wearable structure is devised to tackle the aforementioned concerns in line with the principles and force characteristics of orthotic devices. Initially, an extensive surface area is embraced, employing a semi-enclosed wearable structure to evenly disperse the applied pressure on the leg. Subsequently, the wearable structure encompasses multiple pores, facilitating efficient heat dissipation and sweat evaporation. Moreover, lightweight and flexible materials are employed to lessen the weight of the components without compromising their durability. Lastly, the design adopts a shape that impeccably conforms to the contours of the human skin.

The irregular shape of the human leg surface makes it challenging to directly obtain the surface curvature measurements. However, by utilizing three-dimensional scanning technology, it is possible to acquire spatial point cloud data of the measured leg surface. This data can undergo a series of reconstruction processes to generate a three-dimensional mesh model of the measured object. Subsequently, through 3D reconstruction, a 3D model of the human leg surface can be obtained.

**FIGURE 7:**
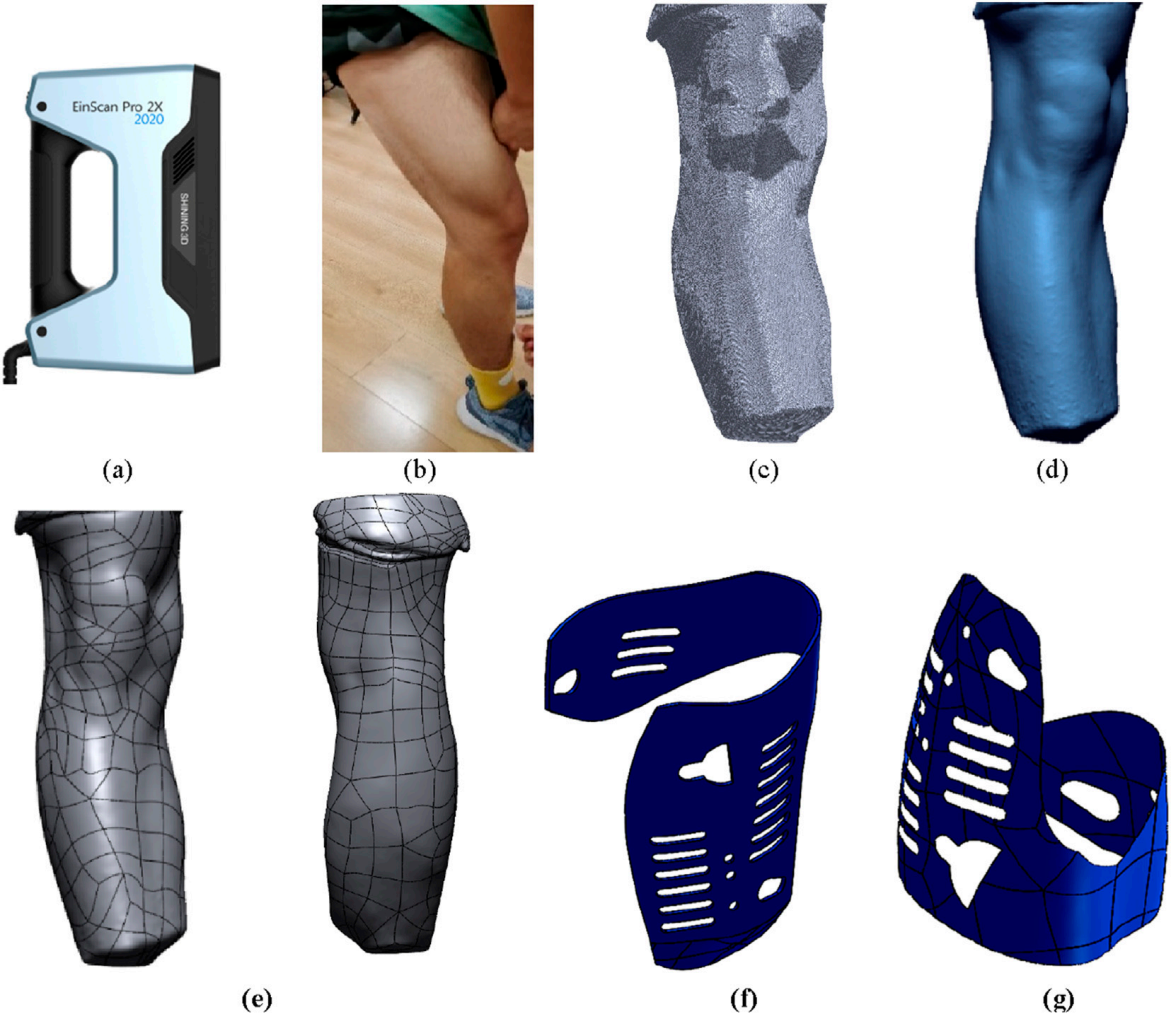
Design and modeling process of wearable structure. **(A)** EinScanPro 2X professional handheld 3D scanner. **(B)** The surface of the human leg. **(C)** The point cloud data. **(D)** The surface model and perform surface construction. **(E)** Surface optimization model. **(F)** The upper semi-enclosed wear structure. **(G)** The lower semi-enclosed wear structure.

**FIGURE 8:**
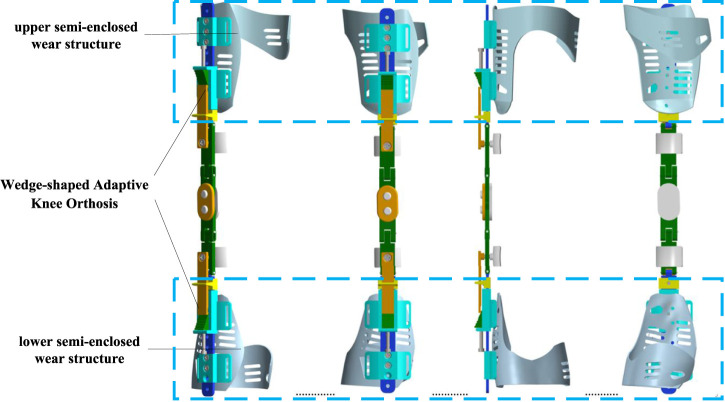
3D view of the knee orthoses with wearing structures.

Using the EinScanPro 2X professional handheld 3D scanner, as shown in Figure 7A, three-dimensional data collection of the surface of the human leg was conducted, as shown in Figure 7B. After the scanning process, the built-in algorithm of the scanner automatically aligns and merges the point cloud data, as shown in Figure 7C. The scanned environmental objects and excess point clouds of the leg were manually removed, and obtained STL data. The data was imported into the 3D modeling software Solidworks to create a surface model and perform surface construction, as shown in Figure 7D. The noise reduction and optimization techniques on the model were also employed. Furthermore, the data was imported into Geomagic Studio software to generate surfaces and exported the reverse model in Step format for forward design purposes, as shown in Figure 7E. Additional optimization was performed on the surface using Geomagic software. When designing the structure for legwear, it is important to ensure that the human joints and areas of movement are not covered by fixtures. Therefore, the part from the scanned model was removed, keeping only the middle part of the leg for designing a semi-enclosed wear structure, as shown in Figures 7F, G. The wear structure model was then 3D printed and assembled with the orthosis structure, resulting in a complete prototype of the wearable orthosis as shown in Figure 8.

### 3.3 Working principle

Through screw transmission, the correction angle of the knee orthosis is finally achieved, so as to provide different degrees of external force to the knee joint and alleviate the pressure on the affected side of the knee joint. Ratchet teeth are distributed on the outside of the toothed wedge blocks, which can engage with the ratchet bar on the lever arm to realize one-way transmission and reverse self-locking of the toothed wedge. Patients can adjust the effect according to their own needs. The knee joint buckling connecting rod with a special structure can not only meet the sagittal motion of the knee joint but also take into account the small range motion of the knee joint on the coronal plane. At the same time, it can compensate for the movement of the orthosis caused by joint movement.

**FIGURE 9:**
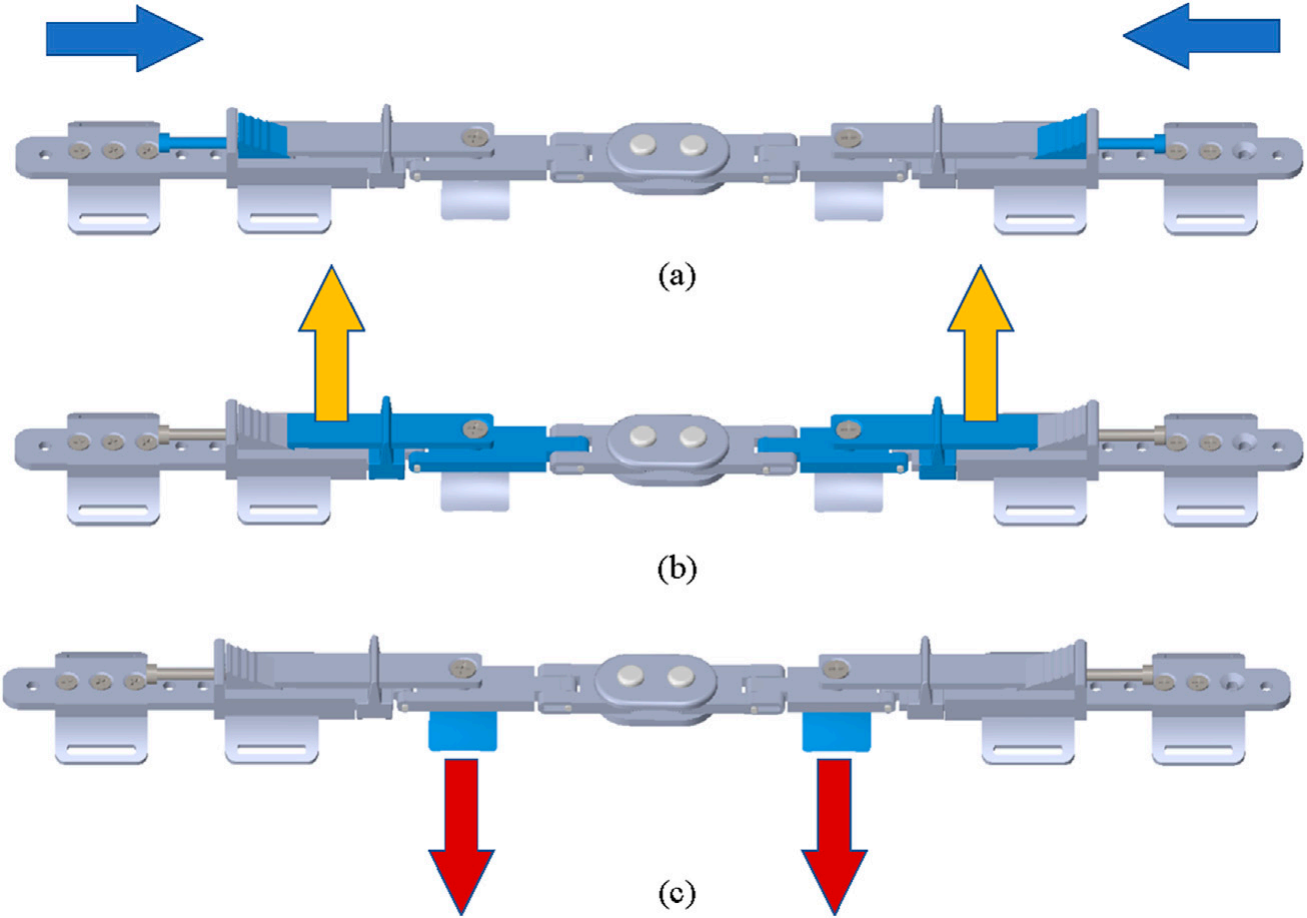
The workflow of new adjustable knee orthosis. **(A)** The adjusting screw drives the toothed wedge block to move towards the inside of the knee. **(B)** The toothed wedge block push the lever arm up to the outside. **(C)** The adjusting force bracket moves to the inside of the leg.

The specific working principle of new adjustable knee orthosis is as follows: 1) Rotate the adjusting screw, which will drive the toothed wedge block to move towards the inside of the knee, as shown in Figure 9A; 2) The toothed wedge block will push the lever arm up to the outside, while the other end of the lever arm will squeeze to the inside of the leg, and the lever arm support rod will also move inward, as shown in Figure 9B; 3) Because the knee adjusting force bracket is fixedly connected with the lever arm and the flexion connecting rod when the lever arm squeezes to the inside of the leg, the adjusting force bracket moves to the inside of the leg, thus transferring the pressure from the lever arm to the inside of the leg, as shown in Figure 9C.

**FIGURE 10:**
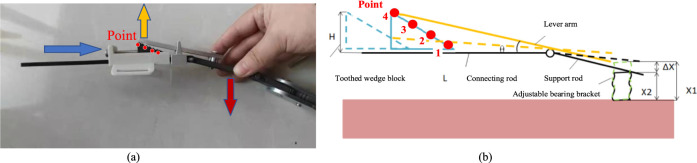
The working principle of new adjustable knee orthosis. **(A)** Diagram of knee orthosis adjustment angle. **(B)** The relationship between the number of screw rotation rings and the adjustment angle. H is the height of toothed wedge block. L is the length of upper connecting rod. ΔX = X1-X2,which is the height difference between each two adjacent adjustment points.

According to human biomechanics, the knee varus angle from 0° to 7°. Therefore, there are four adjustment points distributed on the toothed wedge, as shown in Figure 10A. The wedge block has four serrations distributed on it, and the orthotic device also has four adjustment points. The pitch of the adjusting screw is 0.8 mm. When the adjusting screw rotates 1 circle, it advances 0.8 mm to the medial knee. The relationship between the number of screw rotation rings and the adjustment angle, as shown in Figure 10B can be calculated by trigonometric function, as shown in Table 2.

**TABLE 2:** The empirical formula of the human body parameter.

Adjustment points	H (mm)	L (mm)	Tan θ (ΔH/L)	Number of cycles	Adjustment angle
point1	1.32	53.77	0.025	16	1.5°
point2	3.00	53.49	0.056	21	3°
point3	4.68	53.19	0.087	26	5°
point4	6.36	52.71	0.121	31	7°

## 4 Experiment and functional evaluation

### 4.1 Physical experiment with pressure test

The knee orthosis generates an abduction torque by exerting corresponding pressure on the leg of patients with genu varus, thereby altering the distribution of forces in the medial and lateral compartments of the knee joint. This correction realigns the lower limb mechanical axis, reduces meniscal wear, and alleviates joint pain. Physical prototype testing is essential to validate the structural design and feasibility of the proposed solution before conducting human-wearing experiments. Moreover, direct measurement of forces in the medial and lateral compartments of the knee joint is challenging. Therefore, a portable plantar pressure testing system is designed to objectively evaluate the orthosis by analyzing the pressure values in different regions of the foot, which reflect the effects of wearing on the human body.

**FIGURE 11:**
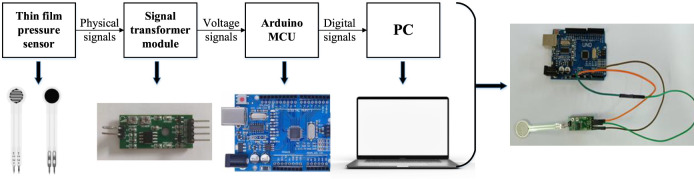
Pressure sensing test system composition diagram.

The experimental system consists of two parts, as shown in Figure 11. The first part is the information acquisition module, which directly collects pressure values and gathers physical pressure signals of the foot’s variations using thin film pressure sensors as sensitive components. Since the thin film pressure sensor outputs millivolt-level analog voltage signals, amplification is required to amplify the collected pressure signals. The second part is the conditioning module for pressure information. The signal is amplified by the decoder and transmitted to a microcontroller, in this case, an Arduino microcontroller. The collected signal is conditioned into a computer-readable digital signal and transmitted to a computer via a data cable for data storage and processing.

**FIGURE 12:**
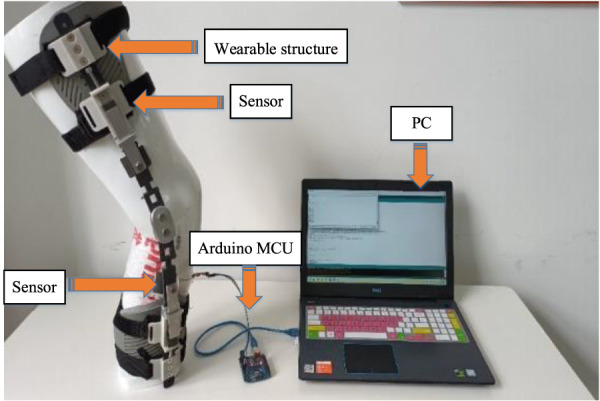
Experimental test with knee orthosis.

The experimental testing device is shown in Figure 12. Using a leg model to replace the human lower limb for testing can effectively simulate the surface curvature of the human leg, enabling close fitting between the orthosis and the lower limb, thus achieving the transmission of forces. However, the main difference between the model and the human leg lies in the definition of materials. The human skeletal structure is enveloped by muscles, which are highly elastic and can be approximated as elastic materials. Therefore, during testing, spring units are clamped between the leg model and the force adjustment bracket of the orthosis. This setup cushions and absorbs the deformations caused by the compression of the leg model by the orthosis’s pressure adjustment module. Also, it effectively transfers the pressure value without any loss or attenuation.

It is used to study the pressure values exerted by the orthosis on the human leg and to determine the range of corrective pressures. Different adjustment levels of the orthosis result in different adjustment angles, and there are also significant differences in the muscle status of individuals. Therefore, during the experiment, four springs with different stiffness coefficients are selected for testing. The parameters of the springs are shown in Table 3. Three measurements are taken for each type of spring.

**TABLE 3:** The parameters of the spring.

Type	Wire diameter (mm)	Mean diameter (mm)	Coil number	Spring stiffness (N/mm)
Spring I	1.3	10	6.5	0.395
Spring II	0.9	4	5	1.845
Spring III	1.7	8	3.5	4.195
Spring IV	2.3	15	2.5	2.985

**FIGURE 13:**
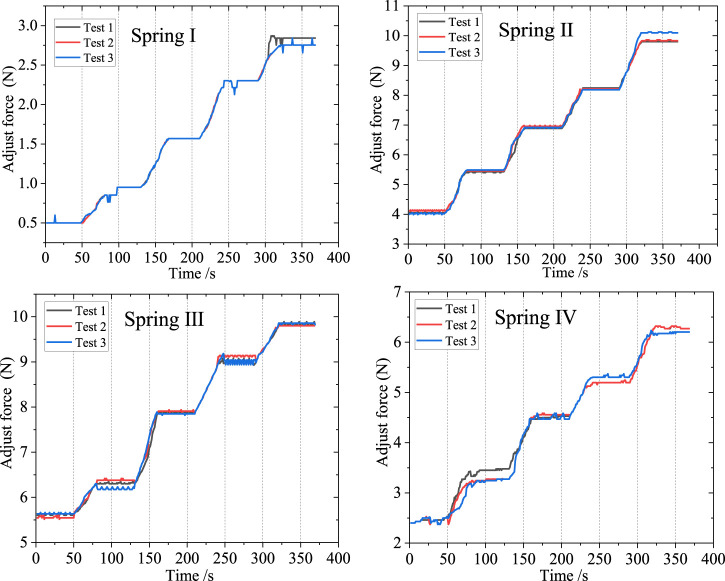
Spring pressure value data curve.

The experimental steps are as follows: 1) Wear the orthosis. 2) Open the Arduino software for serial communication debugging. 3) Calibrate the thin film pressure sensor with known mass weights before testing. 4) Place the thin film pressure sensor at the desired pressure point, ensuring a tight fit. 5) Position and secure the model’s leg vertically. 6) Open the computer again, run the software, and simultaneously adjust the orthosis to different levels. 7) Repeat step 6 three times, retaining three sets of valid experimental data. After each experiment, calibrate the pressure sensor with standard mass block. Adjust the orthosis level as evenly as possible, and try to maintain an equal duration between each adjust level, ensuring that the time intervals for each experiment measurement are equal. The test results were collected and plotted as test curves, as shown in Figure 13.

In this study, four distinct springs characterized by different stiffness coefficients were chosen for experimental evaluation. Experimental data was meticulously collected, and subsequent analysis involved organizing and graphing the resulting force curves. To optimize the interface between the orthosis and the human body, thereby enhancing the effective transmission of corrective loads, a preloading procedure was implemented before any contact-induced deformation. Notably, upon wearing the orthosis and securely fastening the straps, the initial preload forces inherently varied owing to the disparate stiffness properties exhibited by the selected springs.

As the orthosis commenced its operation, accompanied by the continuous adjustment of the gear position during the experimental process, the pressure values correspondingly increased, resulting in an overall stepped curve pattern. Each gear level corresponded to a specific pressure value, aligning with the anticipated design. During the uniform rotational adjustment of the adjusting screw, the triangular block moved downward, and upon entering the corrective gear level, the teeth on the surface of the triangular block engaged with the groove on one end of the lever arm, thereby lifting the lever and causing it to rotate. This rotational movement of the lever arm transferred force through the lever, resulting in a stable corrective force at the knee joint. Consequently, during this phase, the curve exhibited a horizontal segment. Due to the nearly uniform speed of the triangular block’s movement, there were no significant sudden force changes or step-like transitions between adjacent corrective gear levels. Instead, a relatively uniform ascending segment with approximately consistent slope was observed.

The small-scale fluctuations observed in the curve can be attributed to two primary factors. Firstly, these fluctuations arise as a consequence of system friction, encompassing the frictional forces between the triangular block and the track, as well as the friction between the orthosis and the leg. Secondly, these fluctuations originate from vibrations, particularly those induced by the impact between the teeth of the triangular block and the grooves during gear shifting. In terms of the four sets of springs used in the testing, the orthosis can exert a maximum corrective pressure of 10N, which is employed to adjust the forces on the inner and outer sides of the knee joint. Moreover, due to the gradient settings of the gear levels, the orthosis possesses a certain range of adjustment, allowing it to better meet the correction and adjustment needs of patients with habitual arthritis. Additionally, it is possible to select springs that match the individualized requirements of patients, thereby obtaining a broader range of corrective force.

### 4.2 Functional evaluation with plantar pressure test

The effectiveness of the orthosis was evaluated by analyzing the changes in pressure values at eight points on the plantar surface before and after subjects wore the orthosis. This experiment aimed to maintain subjects’ daily habits, ensure ease of wearing during testing, and minimize any impact on flexibility. The shoe insole pressure testing method was employed, wherein an insole with built-in sensors was placed inside the experimental shoes worn during testing. Subjects were instructed to ensure proper contact between their foot soles and the sensor insole after wearing the experimental shoes. The human foot consists of a total of 26 bones. Based on the anatomical structure of the human body this experiment selected eight specific bones for the placement of pressure sensors. These bones include the first metatarsal bone, the second and third metatarsal bones, the fourth and fifth metatarsal bones, the lateral side above the foot, the lateral side below the foot, the medial side of the heel, the middle of the heel, and the lateral side of the heel. These eight areas represent the regions of the foot that experience significant pressure during weight-bearing activities.

**FIGURE 14:**
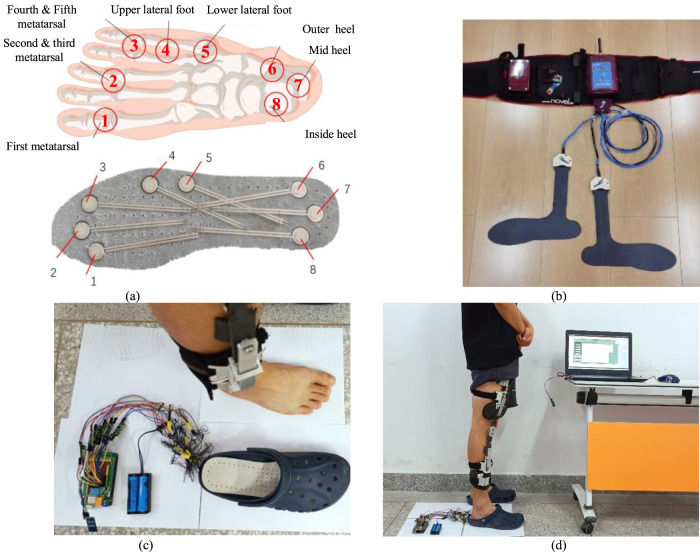
Plantar pressure test experiment with knee orthosis. **(A)** Position of the plantar pressure sensor relative to foot bones. These bones include the first metatarsal bone ①, the second and third metatarsal bones ②, the fourth and fifth metatarsal bones ③, the lateral upper side above the foot ④, the lateral side below the foot ⑤, the outer side of the heel ⑥, the middle of the heel⑦, and the inside of the heel⑧. **(B)** The self-made plantar pressure sensor was used for experimental testing. **(C)** The Novell Pedar insole plantar pressure tester. **(D)** The subject wore knee orthosis and plantar pressure sensor for experimental test with test software.

During the experiment, sensor insoles depicted in Figure 14 were utilized. These insoles were equipped with 8 pressure sensors positioned at various locations. These specific positions on the plantar surface of the foot were accordingly matched with the 8 pressure sensors. The experiment involved a total of 15 subjects, there are 5 subjects for each group, categorized into weight ranges of 60–65 kg, 70–75 kg, and 80–85 kg. Each participant underwent three sets of foot pressure tests, consisting of one test without wearing the orthosis and two tests while wearing the orthosis.

**FIGURE 15:**
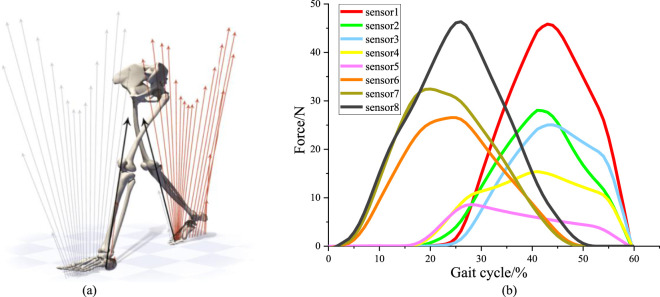
Plantar pressure sensor test experiment. **(A)** Changes of plantar pressure during human walking. **(B)** The force curve within stance phase of right leg depends on the sensors.

To validate the accuracy of the plantar pressure data collected by the self-made device, a commercially available experimental device was used to obtain plantar pressure data as a control. The subjects did not wear orthotics, only wearing plantar pressure insoles for testing. The left foot was tested using a pressure-testing insole from a commercial device, while the right foot was tested using a self-made pressure-testing insole. The variation curve of the measurement values of each pressure sensor with the gait cycle was obtained, as shown in Figure 15.

During the walking state, the soles of both feet sequentially impact the ground, as shown in Figure 15A. Each gait cycle consists of two phases: the stance phase and the swing phase. The stance phase accounts for approximately 60% of the gait cycle and includes three stages: heel strike, foot support, and toe-off. The swing phase accounts for approximately 40% of the gait cycle and includes two stages: leg lift and swing. Therefore, for the pressure curve of a single foot, the pressure between the foot and the ground only acts during the first 60% of the gait cycle (Karimi et al., 2012). Pressure signals from the sensors were collected to plot the pressure curve within stance phase of right leg, as shown in Figure 15B.

**FIGURE 16:**
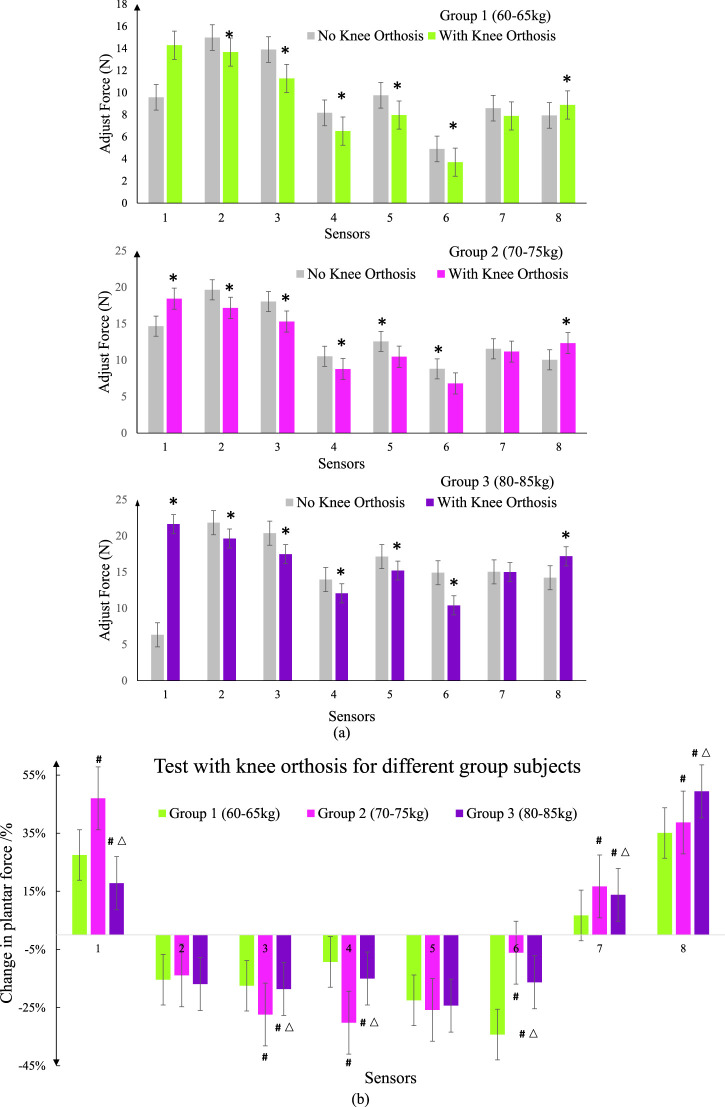
Spring pressure value data curve. **(A)** Compare the test data of self-made equipment with commercial equipment for different group subjects. * represents a significant difference from the No Knee Orthosis group **(B)** Test pressure values with knee orthosis for different group subjects.; # represents a significant difference from the group 1; △ represents a significant difference from the group 2.

The subjects underwent individual experiments for body wearing tests while wearing orthotics. Firstly, the sensors were calibrated to ensure accurate measurements. The sensors were arranged on the insoles and inserted into the designated experimental shoes. The subjects wore these shoes, with the hardware’s connecting wires securely fastened to their legs. During the test, subjects stood still, ensuring their feet were positioned shoulder-width apart. For optimal correction, the orthotic angle was adjusted to its maximum value. Once the sensors received stable signals, measurements were recorded continuously for a duration of 30 s. This entire process was repeated five times, with each subsequent measurement being conducted after a time interval of 3 min. During these intervals, the subjects removed their feet from the experimental shoes, allowing the insoles to return to an unloaded state. Subsequently, the measurements were repeated following the insertion of the orthotics. Finally, the data obtained from each sensor, depicted in Figure 16.

Data differences in the icons are represented by the mean ± standard error (SEM) for each group. Analysis of variance (one-way analysis of variance) and t-test were used to detect whether the difference between groups was significant. Unless otherwise stated, all statistical analyses were performed using Origin (Originlab, United States). *P* < 0.05 was considered statistically significant.

By comparing and calibrating the foot pressure data collected by the homemade device with the foot pressure values obtained from a commercially available experimental apparatus, it was found that the pressure values collected by the sensors of the homemade experimental device are consistent with the data collected by the sensors of the commercial experimental device. Therefore, the data obtained from the foot pressure measurement system can be used for subsequent experimental analysis.

Figures 16A, B present graphical representations of the average data collected from static foot pressure tests conducted on subjects in three different weight ranges. The gray shaded area represents the foot pressure values collected without wearing orthotics, serving as the control group for each experiment. By analyzing the experimental results from the three groups of subjects, it can be observed that after wearing orthotics, there is a significant decrease in pressure values at points 3, 4, 5, and 6 for patients with medial knee instability. Points 3, 4, 5, and 6 on the sensor insole correspond to the fourth to fifth metatarsal heads, the lateral aspect above the foot, the lateral aspect below the foot, and the lateral aspect of the heel respectively. For patients with medial knee instability, due to the deviation of the lower limb alignment, the pressure on the lateral side of the foot is greater compared to that of normal individuals. There is a noticeable increase in pressure values at points 1, 7, and 8, which can be attributed to the effect of the orthotics causing a shift in the body’s center of gravity and adjusting the forces acting on the medial and lateral sides of the knee joint. These results are consistent with the expected outcomes. Comparing the effects of wearing orthotics among the three groups of subjects, the first group exhibited better corrective effects, with a relatively larger decrease in pressure values in the measured area. This can be attributed to the relatively lighter body weight of the subjects in the first group, which makes the corrective effects of the orthotics more pronounced. As body weight increases, the localized adjustment of the force line through the orthotics becomes weaker. Therefore, for patients with medial knee instability, actively reducing body weight is also necessary.

## 5 Discussion and conclusion

### 5.1 Discussion

The knee joint, the largest and most complex joint in the human body, bears significant weight and undergoes frequent movement during human activities. It is also one of the joints prone to injury. The incidence of knee osteoarthritis increases with age. Knee orthoses play a crucial role in mid-stage knee osteoarthritis and post-operative recovery. Through the analysis of existing knee orthoses, it was found that the load-bearing structures of these orthoses primarily use lightweight Aluminum alloys or rigid plastics, reducing overall weight while ensuring adequate support strength. Inner linings commonly consist of rubber and nylon materials, layered to contact the skin, utilizing flexible materials to enhance surface conformity and employing curved designs to minimize contact area, thereby promoting skin breathability and perspiration. The knee orthosis’ fastening mechanisms feature non-slip rubber strips and highly elastic fabrics to prevent sliding and failure during daily use and physical activity. They incorporate buckle devices for quick donning and doffing. The majority of existing products rely on structural wearing to restrict knee joint flexion angles, enhance knee joint support, reduce meniscal load, and alleviate knee joint pain. However, there is a shortage of corrective products for the knee joint coronal plane. Products offering precise graded force adjustment tailored to individual patient needs and rehabilitation course requirements are limited.

Addressing issues such as poor human-machine coordination and discomfort during the wear of existing orthoses, this paper conducts a force analysis of the knee joint. It proposes a four-point force correction scheme and designs an adaptive knee orthosis to address existing product deficiencies and shortcomings. After the structural design and prototype processing test, the expected experimental results were obtained. However, after analysis and demonstration, there are still some problems, which need further research and discussion in the future.

In this paper, a simplified internal and external force model of the knee joint is established, but the structure of the knee joint is complex, and there are many muscles and ligaments related to the movement of the knee joint. Subsequently, the corresponding research can be carried out on the optimization of the mechanical model of the knee joint. Besides, the force line of the human lower limb and the force of orthosis are analyzed, but the influence of coordinated motion between the human lower limb and orthosis is not considered. The next step is to establish a human-machine coupling analysis system, which will have far-reaching significance for the analysis of the interaction between orthosis and the human body and the improvement of the wear effect. The corresponding research will be carried out in the future.

The lower limbs of the human body are accurately scanned and 3D printed for the structural design of orthotics. However, due to individual differences, the universal design is lacking. Although the well-designed wearing structure ensures a good fit between the device and the human body, and the design method is worth popularizing, the traditional bandages and Velcro are still used for binding and pasting, and the fixing methods of the device and the winding methods of the bandage lack depth design. Also, the adjusting mechanism of orthosis is driven by a thread, which realizes the fine adjustment of classification, but the rotation operation of the thread is inconvenient. The later stage should be equipped with a detachable thread torsion lever moment arm to achieve labor-saving and convenient operation. The adjustment range of orthosis should be expanded to the research population, and the limb parameters of special groups such as children should be considered for structural design.

Although this paper verifies that knee orthosis can achieve correction function, human testing needs to recruit more patients with knee varus for testing. Because KOA affects walking speed, range of motion, rhythm, stride length, and increases the adduction moment during walking, these parameters should be fully utilized to measure the performance of orthotics in later clinical trials. The next step will focus on the study of different degrees of KOA patients’ acceptance of orthosis, especially after long-term wear, the impact on human lower limb bones and muscles. It is believed that with the improvement of follow-up research work, knee orthosis will be developed.

Optimization design is a valuable tool for constructing simplified mathematical models, with the typical objective of minimizing the weight of orthotic structures while satisfying specific constraints on wear and fixation, to achieve optimal adjustment forces between the inner and outer compartments of the knee joint. Within orthotic structures, the primary stress-bearing components are the toothed wedges and lever arms. The lever arm, serving as a force transmission mechanism, determines the overall adjustment pressure of the orthosis based on its length and angle. In contrast, the geometric shape of the toothed wedge determines the external pressure applied at each adjustment level. These components interact structurally and influence each other mechanically.

In traditional structural shape optimization design methods, employing finite element techniques facilitates addressing these issues. Utilizing general finite element analysis software enhances design efficiency while meeting specific design requirements. Research focuses on optimizing design parameters of toothed wedges and lever arms through finite element simulation and iterative refinement. Analyzing optimization results identifies structural weaknesses, and determining optimal values for lever arm length and toothed wedge angles to achieve satisfactory design solutions.

For orthotic structural design, the goal of structural optimization is to achieve a safe and economical structure, encompassing the dimensions and shapes of the orthosis. Within allowable structural limits, optimization focuses on adjusting lever arm dimensions (length, width, thickness) and geometric angles of the toothed wedges to ensure high strength while accommodating additional adjustment pressures. Regarding the design of orthotic wearing structures, despite utilizing 3D scanning technology to obtain three-dimensional models of the leg surface, ensuring a snug fit with the body’s leg surface for comfortable wear, the static nature of this fit does not account for dynamic changes in force positions during prolonged wear and walking by patients. Subsequent optimization designs can consider material reduction while maintaining structural stability, incorporating scientifically reasoned heat dissipation hollow structures to facilitate skin cooling and perspiration management.

### 5.2 Conclusion

Knee orthosis plays an important role in the mid-term and postoperative rehabilitation of knee osteoarthritis. However, most of the existing orthoses have problems such as fixed adjustment angle, uncomfortably worn, and interference of walking movement of lower limbs. In this paper, a theoretical model of knee biomechanics is established to determine the relationship between the force of the knee joint in the medial and lateral compartments and the force line of the lower limb, which provides a theoretical basis for the design of auxiliary structures. In order to reduce the knee adduction torque, a knee orthosis was designed based on the four-point force principle. The processing principle was verified by a physical pressure test. The experimental results show that the orthosis can realize step-by-step pressure adjustment and meet the design requirements. The results of the physical stress test show that the orthosis can achieve an angular range adjustment of 0°–7° and can provide a corrective force of 0–10N to the knee joint.

## Data Availability

The raw data supporting the conclusions of this article will be made available by the authors, without undue reservation.

## References

[B1] Aleksandra R. B. Bradley E. M. Stephen F. Cowper-Smith C. D. (2020). Design evaluation of a novel multicompartment unloader knee brace. J. Biomechanical Eng. 142, 0145021–0145028. 10.1115/1.4044818 31523751

[B2] Arazpour M. Ahmadi B. M. Hutchins S. W. Jones R. K. Habibi Babadi M. (2013). Frontal plane corrective ability of a new unloader orthosis for medial compartment of the knee. Prosthetics Orthot. Int. 37 (6), 481–488. 10.1177/0309364613478964 23471227

[B3] Bliddal H. Christensen R. (2009). The treatment and prevention of knee osteoarthritis: a tool for clinical decision-making. Expert Opin. Pharmacother. 10 (11), 1793–1804. 10.1517/14656560903018911 19537998

[B4] Cheng G. H. Younis A. Hajikolaei K. H. Wang G. G. (2012). Trust region based MPS method for global optimization of high dimensional design problems. J. Mech. Des. 137 (2). Aiaa/asme/asce/ahs/asc Structures, Structural Dynamics and Materials Conference Aiaa/asme/ahs Adaptive Structures Conference Aiaa. 10.1115/1.4029219

[B5] Cooke T. D. V. Sled E. A. Scudamore R. A. (2007). Frontal plane knee alignment: a call for standardized measurement. J. Rheumatology 34 (9), 1796–1801. 10.1097/01.bor.0000285007.30493.f9 17787049

[B6] Cynthia H. Fantini P. Wolfgang P. (2009). The effect of valgus bracing on the knee adduction moment during gait and running in male subjects with varus alignment. Clin. Biomech. 25 (1), 70–76. 10.1016/j.clinbiomech.2009.08.010 19758735

[B7] Esrafilian A. Karimi M. T. Eshraghi A. (2012). “Design and evaluation of a new type of knee orthosis to align the mediolateral angle of the knee joint with osteoarthritis,” in Proceedings of 2012 2nd international conference on biomedical engineering and technology (Hong Kong, China: Ed. IACSIT Press), 100–104. 10.1155/2012/104927 PMC3345217 22577565

[B8] Folmar E. Jennings H. Lusardi M. (2020). “Principles of lower extremity orthoses,” in Orthotics and prosthetics in rehabilitation. Four Edition, 220–258. 10.1016/B978-0-323-60913-5.00009-X

[B9] Gaasbeek R. D. A. Groen B. E. Hampsink B. van Heerwaarden R. J. Duysens J. (2007). Valgus bracing in patients with medial compartment osteoarthritis of the knee. Gait and posture 26 (1), 3–10. 10.1016/j.gaitpost.2006.07.007 16962329

[B10] Hangalur G. Bakker R. Tomescu S. Chandrashekar N. (2017). New adjustable unloader knee brace and its effectiveness. J. Med. Devices 12, 1. 10.1115/1.4038439

[B11] Hartofilakidis G. Babis G. C. Lampropoulouadamidou K. (2014). “Treatment options, except total hip replacement: conservative management and osteotomies,” in Congenital hip disease in adults (Milano: Springer). 10.1007/978-88-470-5492-9_5

[B12] Heidari B. (2011). Knee osteoarthritis prevalence, risk factors, pathogenesis and features: Part I. Casp. J. Intern. Med. 2 (2), 205–212. PMC3766936 24024017

[B13] Hunt M. Birmingham T. Bryant D. Jones I. Giffin J. Jenkyn T. (2008). Lateral trunk lean explains variation in dynamic knee joint load in patients with medial compartment knee osteoarthritis. Osteoarthr. and Cartil. 16 (5), 591–599. 10.1016/j.joca.2007.10.017 18206395

[B14] Hunt M. A. Birmingham T. B. Giffin J. R. Jenkyn T. R. (2006). Associations among knee adduction moment, frontal plane ground reaction force, and lever arm during walking in patients with knee osteoarthritis. J. Biomechanics 39 (12), 2213–2220. 10.1016/j.jbiomech.2005.07.002 16168997

[B15] Jackson B. Wluka A. Teichtahl A. Morris M. Cicuttini F. (2004). Reviewing knee osteoarthritis-a biomechanical perspective. J. Sci. Med. Sport 7 (3), 347–357. 10.1016/S1440-2440(04)80030-6 15518300

[B16] Karimi M. T. Esrafilian A. Amiri P. (2012). Design and evaluation of a new type of knee orthosis to align the mediolateral angle of the knee joint with osteoarthritis. Adv. Orthop. 2012, 1–6. 10.1155/2012/104927 PMC3345217 22577565

[B17] Khosravi M. Arazpour M. Saeedi H. Rezaei M. (2019). Design evaluation in novel orthoses for patients with medial knee osteoarthritis. J. Biomed. Phys. and Eng. 9 (6), 719–732. 10.31661/jbpe.v0i0.965 32039103 PMC6943847

[B18] Losina E. Weinstein A. Reichmann W. Burbine S. A. Solomon D. H. Daigle M. E. (2012). Lifetime risk and age at diagnosis of symptomatic knee osteoarthritis in the US. Arthritis Care and Res. 65 (05), 703–711. 10.1002/acr.21898 PMC3886119 23203864

[B19] Louise M. Todd S. Charles G. H. Renner J. B. Tudor G. Koch G. (2008). Lifetime risk of symptomatic knee osteoarthritis. Arthritis rheumatism 59 (9), 1207–1213. 10.1002/art.24021 18759314 PMC4516049

[B20] Masouros S. Bull A. Amis A. (2010). (i) Biomechanics of the knee joint. Orthop. Trauma 24 (2), 84–91. 10.1016/j.mporth.2010.03.005

[B21] Özkaya N. Nordin M. Goldsheyder D. Leger D. (2012). Fundamentals of biomechanics impulse and momentum. New York, NY: Springer, 151–164. Chapter 11. 10.1007/978-1-4614-1150-5

[B22] Pandy M. G. Andriacchi T. P. (2010). Muscle and joint function in human locomotion. Annu. Rev. Biomed. Eng. 12 (1), 401–433. 10.1146/annurev-bioeng-070909-105259 20617942

[B23] Reeves Neil D. Bowling F. L. (2011). Conservative biomechanical strategies for knee osteoarthritis. Nat. Rev. Rheumatol. 7 (2), 113–122. 10.1038/nrrheum.2010.212 21289615

[B24] Rezaei M. Saeedi H. Hajiaghaei B. Khademi Kalantari K. Arazpour M. (2019). Comparison of immediate effect of new knee brace and conventional three-points knee valgus Brace on knee adduction moment and ROM in patients with medial knee osteoarthritis. J. Biomed. Phys. Eng. 9 (02), 1–7. 10.31661/jbpe.v0i0.1013 36059283 PMC9395621

[B25] Safari A. Younis A. Wang G. Lemu H. Dong Z. (2015). Development of a metamodel assisted sampling approach to aerodynamic shape optimization problems. J. Mech. Sci. Technol. 29 (5), 2013–2024. 10.1007/s12206-015-0422-5

[B26] Shelburne B. K. Torry R. M. Steadman R. J. Pandy M. G. (2008). Effects of foot orthoses and valgus bracing on the knee adduction moment and medial joint load during gait. Clin. Biomech. 23 (6), 814–821. 10.1016/j.clinbiomech.2008.02.005 18362043

[B27] Shull P. B. Lurie K. L. Cutkosky M. R. Besier T. F. (2011). Training multi-parameter gaits to reduce the knee adduction moment with data-driven models and haptic feedback. J. Biomechanics 44 (8), 1605–1609. 10.1016/j.jbiomech.2011.03.016 21459384

[B28] Taylor W. R. Heller M. O. Bergmann G. Duda G. N. (2004). Tibio-femoral loading during human gait and stair climbing. J. Orthop. Res. 22 (3), 625–632. 10.1016/j.orthres.2003.09.003 15099644

[B29] Thorp L. E. Wimmer M. A. Block J. A. Moisio K. Shott S. Goker B. (2006). Bone mineral density in the proximal tibia varies as a function of static alignment and knee adduction angular momentum in individuals with medial knee osteoarthritis. Bone 39 (5), 1116–1122. 10.1016/j.bone.2006.05.001 16782419

[B30] Xin Z. Geng L. Bing H. Li H. Zhang L. Liu X. (2021a). Different prevention and treatment strategies for knee osteoarthritis (KOA) with various lower limb exoskeletons – a comprehensive review. Robotica 39 (8), 1345–1367. 10.1017/S0263574720001216

[B31] Xin Z. Yu L. Kai Q. Shuo H. (2021b). A wedge-shaped spine and rack type adaptive knee joint orthosis for lower limbs. Chin. Invent. Pat. ZL202110793209.8.

[B32] Younis A. AlKhatib F. Dong Z. (2022). Optimal motorcycle engine mount design parameter identification using robust optimization algorithms. Algorithms 15 (8), 271–326. 10.3390/a15080271

[B33] Younis A. Dong Z. M. (2010a). Trends, features, and tests of common and recently introduced global optimization methods. Eng. Optim. 42 (8), 691–718. 10.1080/03052150903386674

[B34] Younis A. Dong Z. M. (2010b). Metamodelling and search using space exploration and unimodal region elimination for design optimization. Eng. Optim. 42 (6), 517–533. 10.1080/03052150903325540

[B35] Younis A. Dong Z. M. (2012). Global optimization using mixed surrogates and space elimination in computationally intensive engineering designs. Int. J. Comput. Methods Eng. Sci. Mech. 13 (4), 272–289. 10.1080/15502287.2012.682196

[B36] Younis A. Dong Z. M. (2022). High-fidelity surrogate based multi-objective optimization algorithm. Algorithms 15 (8), 279. 10.3390/A15080279

[B37] Younis A. Dong Z. M. (2024). Adaptive surrogate assisted multi-objective optimization approach for highly nonlinear and complex engineering design problems. Appl. Soft Comput. 150, 111065. 10.1016/J.ASOC.2023.111065

[B38] Younis A. ElBadawy M. (2022). An adaptive sampling and weighted ensemble of surrogate models for high dimensional global optimization problems. Electron. J. Appl. Stat. Analysis 15 (2), 399–420. 10.1285/i20705948v15n2p399

[B39] Younis A. Xu R. Dong Z. M. (2009). Approximated unimodal region elimination based global optimization method for engineering design. Int. J. Prod. Dev. 9 (1), 164–187. 10.1115/DETC2007-34839

